# Proportion and characteristics of screen-detected and non-screen-detected colorectal cancers in Germany

**DOI:** 10.2340/1651-226X.2024.40234

**Published:** 2024-11-24

**Authors:** Michel Hornschuch, Sarina Schwarz, Ulrike Haug

**Affiliations:** a Department of Clinical Epidemiology, Leibniz Institute for Prevention Research and Epidemiology – BIPS, Bremen, Germany; b Faculty of Human and Health Sciences, University of Bremen, Bremen, Germany

**Keywords:** Screening, colonoscopy, fecal occult blood test, Germany

## Abstract

**Background:**

Germany has a long-standing colorectal cancer (CRC) screening offer. We aimed to quantify and characterize screen-detected colorectal cancers (sdCRCs) in Germany.

**Methods:**

We conducted a cross-sectional study based on a healthcare database covering ~20% of the German population; we included CRC patients aged ≥ 55 years diagnosed in 2010–2018. Patients with a screening colonoscopy or a fecal occult blood test followed by colonoscopy within 180 days before diagnosis were classified as sdCRCs and compared to non-sdCRCs regarding age, stage and comorbidities.

**Results:**

In 2018, 25% of male and 22% of female CRC patients were screen-detected. Regarding characteristics of all included CRC cases (*N* = 82,538), sdCRC patients were younger than non-sdCRCs (average difference men / women: 2.6 / 4.4 years). The proportion of advanced CRC among sdCRCs and non-sdCRCs, respectively, was 33 and 42% in women (men: 36 and 45%). Severe comorbidities were more prevalent in non-sdCRCs compared to sdCRCs (e.g. in male / female patients aged 65–74: 35% vs. 27% / 26% vs. 19%). Prevalences of hypertension and obesity were similar in both groups.

**Interpretation:**

Our study suggests that about one fourth of CRCs in Germany are screen-detected. Among patients with non-sdCRC, not only advanced stage but also severe comorbidity was more common than in sdCRCs.

## Introduction

Colorectal cancer (CRC) is among the most common cancers (60,000 new cases per year) and cause of cancer death (24,000 deaths per year) in Germany [1]. The age-standardized CRC incidence decreased by 22% in men and by 26% in women between 2000 and 2016, and the age-standardized CRC mortality decreased by 36% in men and by 41% in women between 2000 and 2018 [2].

Germany has a long-standing CRC screening offer. The fecal occult blood test (FOBT) has been offered since 1977 and since 2002, screening colonoscopy has additionally been offered to persons aged 55 onwards in parallel to FOBT offered from age 50. There is no upper age limit for CRC screening in Germany [3]. In April 2017, the guaiac FOBT was replaced by quantitative fecal immunochemical testing for hemoglobin (FIT) [4]. In 2019, the opportunistic CRC screening program was converted into an organized CRC screening program [5]. Monitoring of a CRC screening program requires indicators such as the participation rate and the detection rate [6]. Information on the proportion of screen-detected CRCs at the population level is also useful for interpreting patterns and trends in CRC survival given that patients with screen-detected colorectal cancers (sdCRCs) are expected to have a better prognosis compared to patients with non-sdCRCs [7–10]. Furthermore, characterizing patients with sdCRCs compared to patients with non-sdCRCs may be helpful to identify subgroups in which it is important to better inform about CRC screening. To the best of our knowledge, from Germany there is only one study quantifying the proportion of sdCRCs [7] and no study characterizing sdCRCs compared to non-sdCRCs according to comorbidities.

To fill this gap, we aimed to estimate the proportion of sdCRCs in Germany based on health claims data and compare sdCRCs and non-sdCRCs regarding sex, age, stage (advanced vs. non-advanced), comorbidities and regional socioeconomic deprivation.

## Methods

### Study design, setting and participants

We conducted a cross-sectional study in Germany including patients with incident CRC diagnosed between 2010 and 2018 aged ≥55 years at diagnosis. We excluded patients with prevalent CRC. As we used claims data (see ‘data source’) we also applied an exclusion criterion to ensure a sufficiently long pre-observation period for the assessment of variables before CRC diagnosis. Specifically, we excluded patients not continuously insured for at least 3 years before CRC diagnosis. Furthermore, we excluded patients with inconsistent or missing information on age or sex (Supplementary Figure 1).

### Data source

We conducted this study using the German Pharmacoepidemiological Research Database (GePaRD), which is based on claims data from four statutory health insurance providers in Germany and currently includes information on approximately 25 million persons who have been insured with one of the participating providers since 2004 or later [11, 12]. In addition to demographic data, GePaRD contains information on drug dispensations as well as outpatient (i.e. from general practitioners and specialists) and inpatient services and diagnoses. Per data year, there is information on approximately 20% of the general population and all geographical regions of Germany are represented. In Germany, about 90% of the population are covered by statutory health insurance providers. The German health insurance system is characterized by a uniform access to all levels of care. Persons with no income are also covered by statutory health insurances in Germany. Membership in statutory health insurance is compulsory but there are exceptions, e.g. for persons with a very high income and for civil servants.

### Definition of sdCRCs

We defined sdCRCs based on the utilization of screening examinations. In GePaRD, information on the use of colonoscopy and FOBT, including the date of the procedure, is obtained based on codes of the Operations and Procedure Coding System (OPS) and/or the German Uniform Assessment Standard (EBM). For both colonoscopy and FOBT, a distinction can be made between screening and diagnostic purpose, as there are different reimbursement codes for these procedures. Screening examinations conducted within 6 months before CRC diagnosis were considered to be related to CRC diagnosis, in line with the World Endoscopy Organisation Consensus Statement [13]. Specifically, patients with A) a screening colonoscopy or B) an FOBT followed by colonoscopy within 180 days before CRC diagnosis or in the same calendar quarter of CRC diagnosis were classified as sdCRCs. All other patients were classified as non-sdCRCs. In German claims data, the time unit ‘calendar quarter’ is relevant because outpatient diagnoses are coded on a quarterly basis.

### Variables

To identify incident CRC cases in GePaRD, we used a previously developed algorithm [14]. In a first step, the algorithm identifies patients with at least inpatient diagnosis codes, which have a high validity. To avoid misclassification, persons who had exclusively outpatient diagnosis codes of CRC (second step) were only classified as CRC cases if additional criteria (such as regular surveillance examinations according to guidelines handling low-risk pT1 CRCs) were fulfilled. To identify prevalent CRC cases (for exclusion) and distinguish them from incident CRCs, any codes indicating prevalent CRC during the pre-observation period were considered, taking also into account status-post diagnosis codes and codes indicating follow-up care in CRC survivors. The algorithm also classifies CRC diagnoses into advanced and non-advanced considering diagnoses codes for distant metastases and affected lymph nodes as well as treatment used only for advanced stages [14, 15]. The category ‘advanced’ corresponds to TNM stage III–IV (affected lymph nodes or distant metastasis) and the category ‘non-advanced’ corresponds to TNM stage I–II (no affected lymph node, no distant metastasis; information on T-stage is not available in claims data, i.e. no distinction between TNM I and II is possible). Furthermore, the algorithm classifies CRCs into proximal versus distal to the splenic flexure. The CRC incidence, the stage distribution (advanced vs. non-advanced) and the distribution by location (distal vs. proximal) determined based on this algorithm has been shown to agree very well with cancer registry data from the whole of Germany [14].

The prevalence of comorbidities (e.g. treated coronary heart diseases, COPD, dementia) was assessed based on algorithms using data from the two calendar years prior to CRC diagnosis. For sdCRCs, we also assessed whether there were any codes for gastrointestinal symptoms or certain diagnoses that may be related to gastrointestinal symptoms (e.g. Crohn’s disease and/or ulcerative colitis, acute abdominal pain, faecal abnormalities). We assessed the coding of these conditions in the same quarter and in the quarter before the screening colonoscopy or the FOBT. For chronic conditions such as inflammatory bowel disease, we also considered codes recorded earlier.

In order to estimate the regional socioeconomic status, we linked the patients’ district of residence with the German Index of Socioeconomic Deprivation (GISD) developed by the Robert Koch Institute (RKI) as previously described [16]. Based on the GISD, the districts were grouped into three categories (low, middle, high). The category ‘low’, for example, means low deprivation, i.e. it comprises districts with a population that tends to have a higher socioeconomic status.

### Data analysis

We used descriptive analyses to describe all included CRC patients as well as subgroups defined based on the mode of detection (sdCRCs vs. non-sdCRCs) regarding sociodemographic characteristics, stage distribution and comorbidity. We stratified the analyses by sex and partly also by age group. Furthermore, we stratified some of the analyses by year of diagnosis, putting a special focus on 2018 as this was the first year with full coverage of FIT. As compared to the guaiac FOBT, we assume that FIT is more completely captured in claims data given the higher cost and the additional codes from laboratories. For sdCRCs, we assessed the proportion with codes indicating gastrointestinal symptoms or selected diagnoses (see above) to estimate the proportion of truly asymptomatic patients. Finally, we also assessed the proportion of sdCRCs separately for CRCs in the distal versus the proximal colon.

The analyses were carried out with the statistical software SAS 9.4 (SAS Institute, Inc., Cary, North Carolina, USA).

## Results

As shown in Table 1, we included 82,538 CRC patients overall (49% female). The mean age at diagnosis was 72.3 years in men and 73.7 years in women. About 40% of all CRCs were diagnosed at an advanced stage (men: 43%, women: 41%).

**Table 1:** Characteristics of included CRC patients diagnosed between 2010 and 2018.

	Total	Men	Women
**Total^a^, *n* (%)**	82,538 (100.0)	41,922 (100.0)	40,616 (100.0)
**Age at diagnosis, years**			
Mean (SD)	73.0 (9.4)	72.3 (8.9)	73.7 (9.7)
Median [Q1; Q3]	73.0 [66.0; 80.0]	73.0 [66.0; 79.0]	74.0 [66.0; 81.0]
55–59, *n* (%)	7,923 (9.6)	4,033 (9.6)	3,890 (9.6)
60–64, *n* (%)	9,483 (11.5)	5,134 (12.2)	4,349 (10.7)
65–69, *n* (%)	11,980 (14.5)	6,572 (15.7)	5,408 (13.3)
70–74, *n* (%)	15,803 (19.1)	8,518 (20.3)	7,285 (17.9)
75–79, *n* (%)	16,201 (19.6)	8,517 (20.3)	7,684 (18.9)
80–84, *n* (%)	11,386 (13.8)	5,431 (13.0)	5,955 (14.7)
≥ 85, *n* (%)	9,762 (11.8)	3,717 (8.9)	6,045 (14.9)
**Year at diagnosis, *n* (%)**			
2010, *n* (%)	9,000 (10.9)	4,555 (10.9)	4,445 (10.9)
2011, *n* (%)	8,858 (10.7)	4,550 (10.9)	4,308 (10.6)
2012, *n* (%)	8,498 (10.3)	4,263 (10.2)	4,235 (10.4)
2013, *n* (%)	9,030 (10.9)	4,666 (11.1)	4,364 (10.7)
2014, *n* (%)	9,113 (11.0)	4,595 (11.0)	4,518 (11.1)
2015, *n* (%)	9,375 (11.4)	4,805 (11.5)	4,570 (11.3)
2016, *n* (%)	9,379 (11.4)	4,783 (11.4)	4,596 (11.3)
2017, *n* (%)	9,703 (11.8)	4,881 (11.6)	4,822 (11.9)
2018, *n* (%)	9,582 (11.6)	4,824 (11.5)	4,758 (11.7)
**Stage at diagnosis, *n* (%)**			
Advanced, *n* (%)	34,733 (42.1)	18,120 (43.2)	16,613 (40.9)
**Tumor location^b^, *n* (%)**			
Distal, *n* (%)	44,485 (53.9)	25,087 (59.8)	19,398 (47.8)
Proximal, *n* (%)	28,536 (34.6)	12,774 (30.5)	15,762 (38.8)
Both/Unknown, *n* (%)	9,517 (11.5)	4,061 (9.7)	5,456 (13.4)
**GISD of the district of residence^c^, *n* (%)**			
Low, *n* (%)	19,040 (23.1)	9,412 (22.5)	9,628 (23.7)
Middle, *n* (%)	51,863 (62.8)	26,185 (62.5)	25,678 (63.2)
High, *n* (%)	11,495 (13.9)	6,240 (14.9)	5,255 (12.9)

CRC: colorectal cancer; GISD: German Index of Socioeconomic Deprivation; Q1: first quartile, Q3: third quartile; SD: standard deviation; sdCRC: screen-detected colorectal cancer.

a In one of the health insurances providing data of about 6 million insured persons to GePaRD, the proportion of women 50 years old or older is substantially higher as compared to the general population. This explains why the proportion of female CRC patients among all CRC patients is higher than the proportion reported by cancer registries.

b Affected lymph nodes or distant metastases.

c Missing information on GISD: ≤0.2% of all patients.

**Figure 1:**
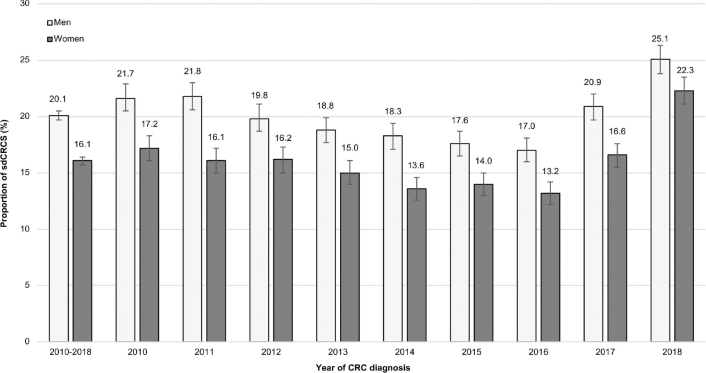
Proportion (including 95% confidence intervals) of screen-detected colorectal cancers (sdCRCs) in men and women overall and stratified by year of colorectal cancer (CRC) diagnosis.

**Figure 2:**
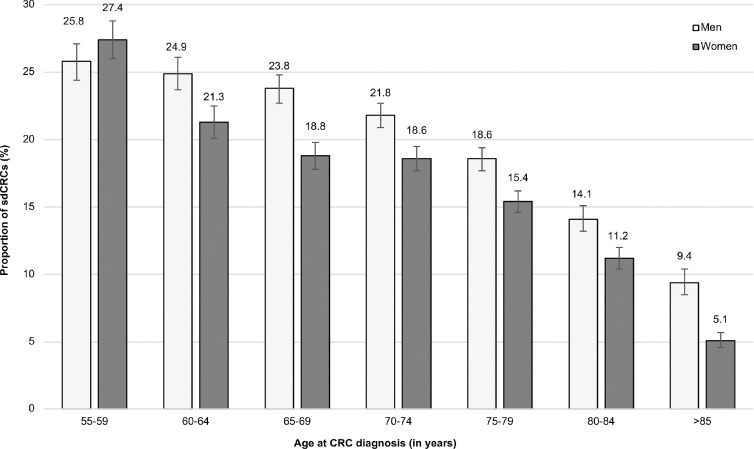
Proportion (including 95% confidence intervals) of screen-detected colorectal cancers (sdCRCs) in men and women according to age at CRC diagnosis.

In 2018, the proportion of sdCRCs was 25% in men and 22% in women. Across all years, the proportion was 20% in men and 16% in women (Figure 1). As shown in Figure 2, the proportion of sdCRCs decreased with age. In men, it was 24–26% in age groups 55–69 years, 19–22% in age groups 70–79 years, 14% in age group 80–84 and 9% in age group ≥85 years. The mean age of male patients with sdCRCs and non-sdCRC was 70.2 years and 72.8 years, respectively (women: 70.0 years and 74.4 years, respectively). The distribution of patients regarding the socioeconomic deprivation of their district of residence was similar in sdCRCs and non-sdCRCs, both in men and women (Table 2).

**Table 2:** Distribution of male and female CRC patients regarding the deprivation index (GISD) of their district of residence stratified by mode of CRC detection.

	Men				Women			
	sdCRCs		non-sdCRCs		sdCRCs		non-sdCRCs	
	%	95% CI	%	95% CI	%	95%	%	95% CI
**GISD of the district of residence^a^**								
Low	23.8	22.9–24.7	22.1	21.7–22.6	24.1	23.1–25.1	23.6	23.2–24.1
Middle	61.9	61.0–63.0	62.6	62.2–63.3	62.0	60.9–63.3	63.4	63.0–64.0
High	14.2	13.4–15.0	15.1	14.7–15.5	13.8	13.0–14.6	12.8	12.4–13.2

CI: confidence interval; CRC: colorectal cancer; GISD: German Index of Socioeconomic Deprivation; sdCRC: screen-detected colorectal cancer.

a Missing GISD: ≤0.2% of all patients.

**Figure 3:**
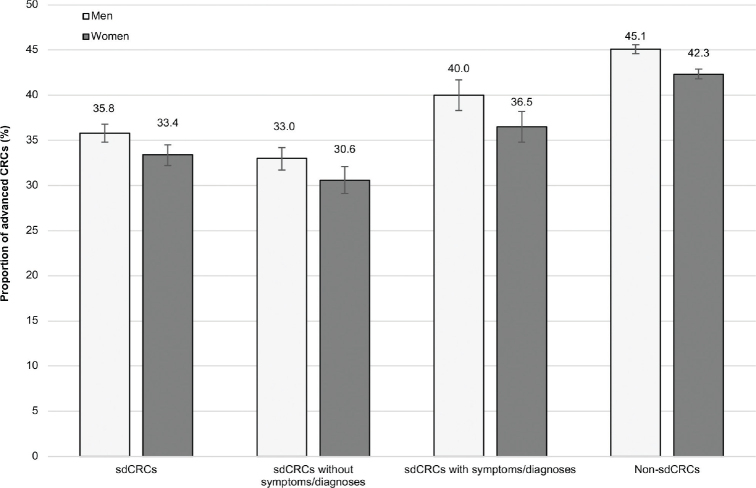
Proportion (including 95% confidence intervals) of advanced colorectal cancers (CRCs) among all screen-detected colorectal cancers (sdCRCs), among sdCRCs with codes for symptoms/ selected diagnoses,^a^ among those without such codes and among non-sdCRCs. ^a^sdCRCs with codes indicating gastrointestinal symptoms (e.g. acute abdominal pain, fecal abnormalities) or with codes for selected diagnoses that may cause gastrointestinal symptoms (e.g. chronic inflammatory bowel disease).

Regarding stage distribution, the proportion of advanced CRC was 36% in male patients with sdCRCs and 45% in those with non-sdCRCs (women: 33 and 42%, respectively). The proportions were similar when we considered only CRCs diagnosed in 2018 (difference in point estimates ≤ 2 percentage points, see Supplementary Figure 2). When restricting the sdCRC cases to those without any codes for gastrointestinal symptoms or selected diagnoses that may cause gastrointestinal symptoms (see prevalences described in Supplementary Table 1), the proportion of advanced CRCs decreased to 33% in men and 31% in women (Figure 3). Additional stratification by age showed similar results (Supplementary Table 2). In the analysis by tumor location, the proportion of sdCRCs was 16% in the proximal colon and 20% in the distal colon (Supplementary Table 3).

**Figure 4:**
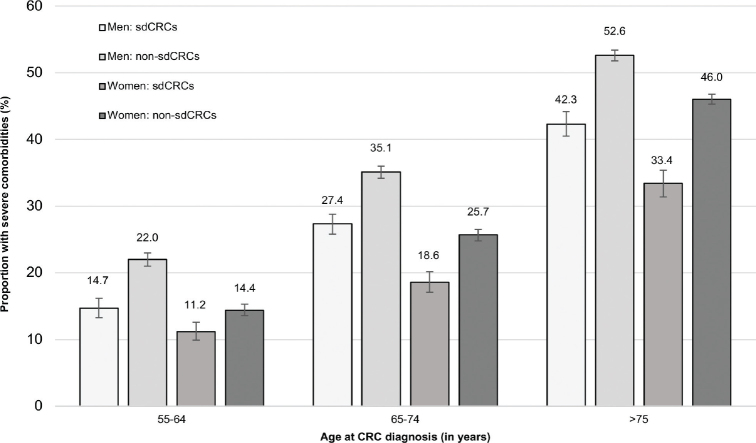
Proportion (including 95% confidence intervals) of patients with at least one severe comorbidity^a^ among men and women with screen-detected colorectal cancers (sdCRCs) compared to those with non-sdCRCs, stratified by age group. ^a^Comorbidities considered here: coronary heart disease, chronic heart failure, acute myocardial infarction, acute stroke, COPD, severe liver disease, end-stage renal disease, diabetes with end-organ damage, immunosuppressive therapy, HIV-therapy, hemiplegia, dementia.

As shown in Figure 4, in all age groups and in both sexes, the proportion of patients with at least one of the severe comorbidities considered in our study was 24–50% higher among patients with non-sdCRCs as compared to patients with sdCRCs. For example, in age group 65–74 years, this proportion was 35% in non-sdCRCs and 27% in sdCRCs (women: 26 and 19%, respectively). The prevalence of other comorbidities such as obesity and the use of antihypertensive or lipid-modifying drugs was similar in patients with non-sdCRC and those with sdCRC (Supplementary Table 4).

## Discussion

Our study, which is the first to provide detailed information on sdCRCs in Germany, suggests that about one fourth of CRCs occurring in patients aged 55 years or older in Germany are currently detected in the context of a screening examination. The large sample size of our study and the comprehensive information available in claims data facilitated description and comparison of sdCRCs and non-sdCRCs regarding factors such as sex, age, comorbidities and regional socioeconomic deprivation. Even though there is no upper age limit for CRC screening in Germany, we found a marked decrease in the proportion of sdCRCs with age. Across all age and sex groups, the proportion of patients with severe comorbidity was 24–50% higher among patients with non-sdCRCs as compared to patients with sdCRCs. Based on regional deprivation indices, we found no relevant disparities regarding the chance of screen-detection of CRCs in Germany.

To the best of our knowledge, the proportion of sdCRCs in Germany has only been reported in the context of one case-control study [7]. This study included 2,450 CRC patients aged 50–79 years recruited between 2003 and 2010. The overall proportion of sdCRCs among persons with CRCs reported in this study (~21%) was in a similar range as the proportion observed in our study in 2010 (women: 22%, men: 17%). The age patterns regarding the proportion of sdCRCs were different: Unlike in our study, the proportions were more or less similar across age groups (50–59: 19%, 60–69: 22%, 70–79: 21%). However, unlike in our study, volunteer bias could have distorted the age pattern of sdCRCs in this study. Regarding our results on other characteristics of sdCRCs and non-sdCRCs there is no study from Germany to which we can compare our findings.

As regards the comparison with other European countries, the proportion of sdCRCs observed in our study agrees well with reports from some other countries but there is variation across Europe. In a study based on medical records and screening databases including patients aged 50–74 years from nine European countries, the overall proportion of sdCRCs was below or around ~20% in Belgium, England, France, Ireland, and Italy. In the Netherlands, Slovenia, Spain, and Denmark, a higher proportion of sdCRCs (~30–40%) was observed [17].

The proportion of sdCRCs is in part influenced by the participation rate in CRC screening. In a previous study assessing the 10-year prevalence of screening colonoscopy, we found an age gradient (e.g. 23% in age group 60–64 years vs. 15% in age group 80–84 years) and slightly lower prevalences in women [18], which is in line with the age and sex patterns observed for the proportion of sdCRCs in this study. Also participation in biennial FOBT decreases with age. For example, in 2018/2019 it was reported to be 15% in men aged 60–64 (women: 22%) versus 10% in men aged 80+ (women: 13%) [19]. The higher FOBT uptake in women than in men is not reflected by the proportion of sdCRCs, but it has to be noticed that this proportion is not only influenced by screening participation. Also, the background prevalence of diagnostic colonoscopy plays a role as it increases the likelihood of detection outside the screening program. The 10-year prevalence of diagnostic colonoscopy is generally high in Germany and slightly higher in women than in men (e.g. 24% vs. 21% in age group 60–64) [18]. The proportion of sdCRCs could also be affected by the quality of colonoscopy even though the miss rate for CRC is expected to be low [20]. Irrespective of the determinants, it is important to observe that the proportion of sdCRCs should not be used to assess the performance of a CRC screening program. CRC screening also aims to reduce CRC incidence by detecting and removing precursor lesions, which is not reflected by the proportion of sdCRCs.

The higher proportion of patients with severe comorbidities among non-sdCRCs compared to sdCRCs observed in our study may partly reflect the self-selection of healthier persons to screening. This self-selection has often been discussed in the context of the so-called healthy screening bias [21]. Interestingly, we found no difference between sdCRC and non-sdCRC patients regarding the proportion using antihypertensive or lipid-modifying drugs. There were also other studies suggesting that the use of these drugs indicates a health-seeking behavior [22]. In this way, the large group of patients using these drugs should be distinguished from patients who actually developed severe cardiovascular comorbidities. From a clinical perspective, it is problematic that patients with severe comorbidity are less often screen-detected. As demonstrated in our study, this also means that these patients are more often diagnosed at an advanced stage, which would require intense therapy. The higher risk of side effects among comorbid patients and the fact that there are less treatment options due to contraindications may explain why survival in CRC patients with comorbidities is worse compared to patients without comorbidities [23]. Thus, when comparing absolute survival between sdCRC and non-sdCRCs not only the stage distribution but also the prevalence of severe comorbidities should be considered in the interpretation of potential differences.

Our study suggests that the proportion of sdCRCs in Germany is similar across regions with different socioeconomic deprivation indices in Germany. These results may add to the interpretation of the studies by Jansen et al. published in 2014, 2020 and 2021 who used cancer registry data to determine the association between regional socioeconomic inequalities and survival of CRC patients in Germany [24–26]. In all studies, the 5-year survival in CRC patients living in the most deprived areas was worse as compared to patients living in less deprived areas. It was hypothesized that screen-detection could partly explain this difference. While the studies by Jansen et al. lacked information on screen-detection, our study does not support this hypothesis.

The difference in the proportion of sdCRCs between men and women observed in our study should not be overinterpreted. Even though the sex difference was larger between 2010 and 2017, the difference was only 2.8 percentage points in 2018 (men: 25.1%; women: 22.3%). After guaiac FOBT has been replaced by FIT in April 2017 [4], 2018 was the first year with full coverage of FIT by health insurances. Earlier, FIT distributed by physicians and self-paid by patients (i.e. not captured in claims data) may have led to a certain proportion of sdCRCs misclassified as non-sdCRCs. Given the role of gynecologists in CRC screening in Germany, this may have been particularly relevant among women and thus have led to an overestimation of sex differences in the proportion of sdCRCs.

Our study has specific strengths and limitations. Given the nature of our database, the analyses are free of recall and non-responder bias. Furthermore, unlike cancer registries, we had information to assess comorbidities and to estimate the proportion of CRC cases detected in the context of a screening examination that might not have been asymptomatic. Although GePaRD does not have full population coverage (~20% of the German population are included in GePaRD), CRC incidence and the proportion of advanced stages have been shown to agree very well with cancer registry data from the whole of Germany [14]. This indicates that the population in GePaRD is representative regarding the prevalence of CRC risk and preventive factors including the uptake of colonoscopy. It also supports the validity of the case definition we used for CRC. As mentioned above, the completeness of capturing fecal occult blood testing in claims data may have changed during the study period, which we considered by conducting analyses stratified by the year of diagnosis. Reassuringly, the differences between 2018 and earlier years were not very large, suggesting that under-ascertainment of fecal occult blood tests between 2010 and 2017 was not a major issue. Similar to prior studies on CRC survival, we assessed socioeconomic differences based on the regional deprivation index on the district level. It is uncertain whether a more precise information on the socioeconomic status would show disparities that we could not detect in our study. On the other hand, we do not expect disparities in this regard as there is consistent evidence from various other studies showing no or only very little differences in the uptake of CRC screening according to socioeconomic status in Germany [18, 27].

In conclusion, our study suggests that about one fourth of CRCs in Germany are screen-detected. Apart from the more favorable stage distribution among patients with sdCRC, also the lower prevalence of severe comorbidities may contribute to an advantage in absolute survival compared to patients with non-sdCRC.

## Supplementary Material

Proportion and characteristics of screen-detected and non-screen-detected colorectal cancers in Germany

## Data Availability

As we are not the owners of the data we are not legally entitled to grant access to the data of the GePaRD. In accordance with German data protection regulations, access to the data is granted only to BIPS employees on the BIPS premises and in the context of approved research projects. Third parties may only access the data in cooperation with BIPS and after signing an agreement for guest researchers at BIPS.
