# Supplementary material for: Proportion and characteristics of screen-detected and non-screen-detected colorectal cancers in Germany
Source: Acta Oncol. 2024 Nov 24;63:40234. doi: 10.2340/1651-226X.2024.40234 (PMC11609877; doi:10.2340/1651-226X.2024.40234)
Supplement: Proportion and characteristics of screen-detected and non-screen-detected colorectal cancers in Germany [file AO-63-40234-s1.pdf]

Supplementary material

Supplementary Figure 1. Flow chart illustrating the inclusion of study participants.

Supplementary Figure 2. Proportion (including 95% confidence intervals) of advanced CRCs among all sdCRCs, among sdCRCs with codes for symptoms/ selected diagnoses, among those without such codes and among non-sdCRCs in year 2018.

Supplementary Table 1. Proportion of sdCRCs with codes indicating symptoms or relevant diagnoses among men and women.

Supplementary Table 2. Proportion of advanced CRCs among all sdCRCs, among sdCRCs with codes for symptoms/ selected diagnoses, among those without such codes and among non-sdCRCs, stratified by age group.

Supplementary Table 3. Proportion of sdCRCs stratified by tumor localization.

Supplementary Table 4. Proportion of patients with at least one comorbidity among men and women with sdCRCs compared to those with non-sdCRCs, stratified by age group (the age distribution within age groups is described in Supplementary Table 4).

Supplementary Table 5. Age distribution of men and women with sdCRCs compared to non-sdCRCs.

Supplementary Figure 1. Flow chart illustrating the inclusion of study participants.

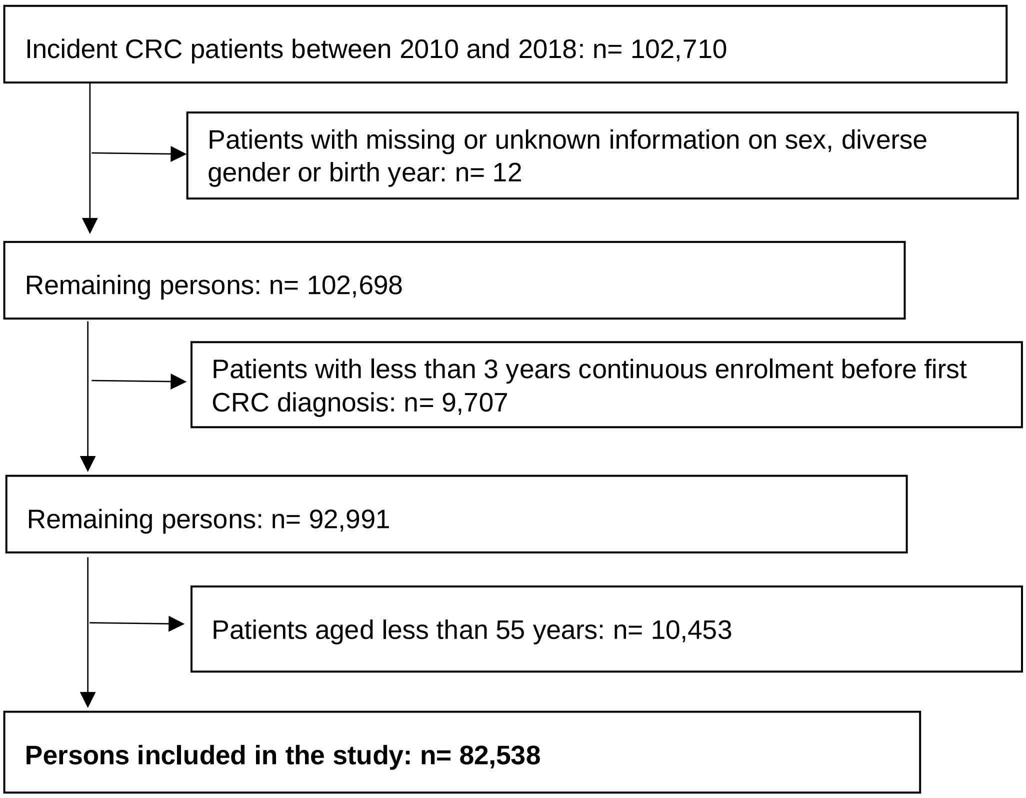

Supplementary Figure 2. Proportion of advanced CRCs among all sdCRCs, among sdCRCs with codes for symptoms/ selected diagnoses[1], among those without such codes and among non-sdCRCs in year 2018.

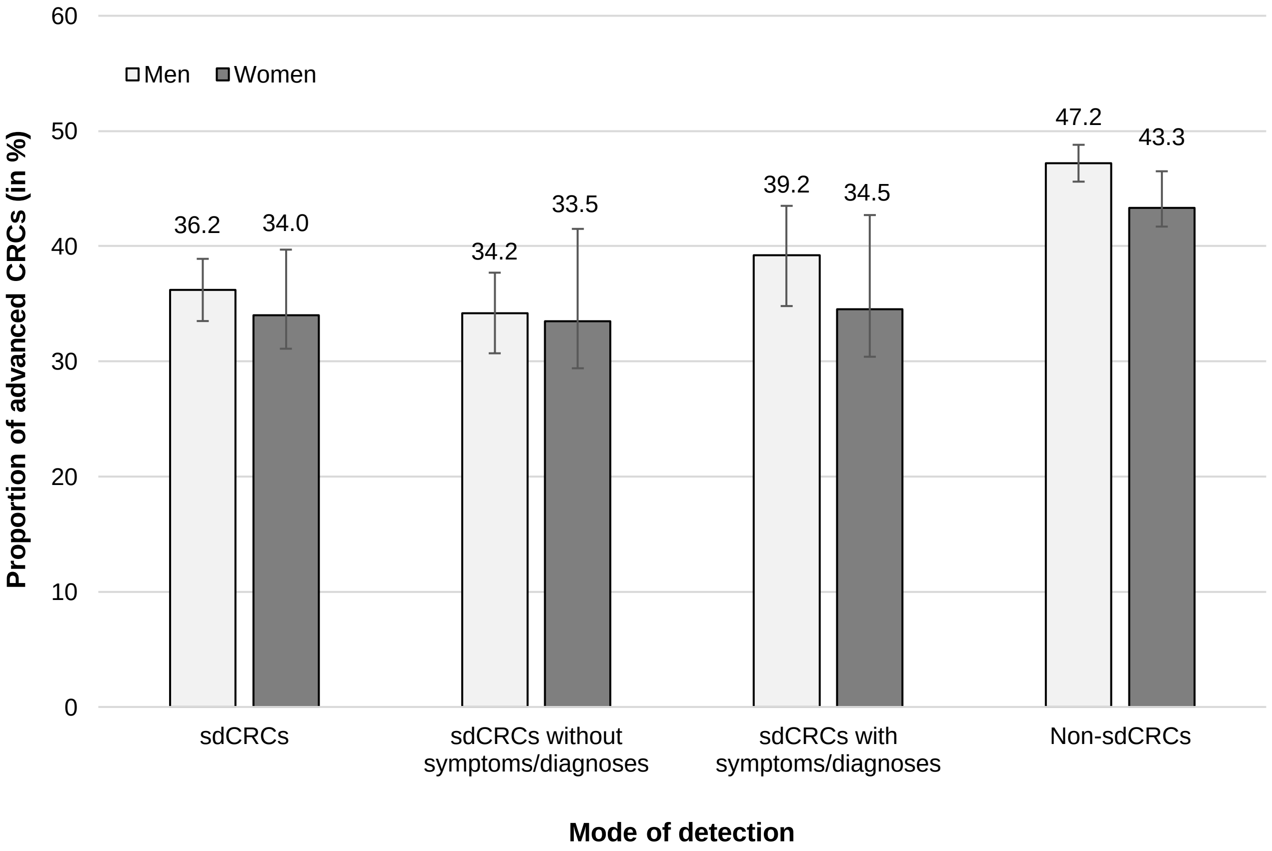

[1]sCRCs with codes indicating symptoms or relevant diagnoses (e.g. Crohn's diseases and/or ulcerative colitis, acute abdominal pain, faecal abnormalities) in the same quarter or before screening colonoscopy/FOBT.

Supplementary Table 1. Proportion of sdCRCs with codes indicating symptoms or relevant diagnoses / relevant other codes among men and women (regarding codes for symptoms, please note that it is not clear whether the symptoms were present at the time of the screening examination).

	**Men**	**Women**
	**n (%)**	**n (%)**
**All CRCs**	8,432 (100.0)	6,519 (100.0)
**Symptoms and diagnosis**		
Crohn's disease[1] and/or ulcerative colitis	100 (1.2)	111 (1.7)
Anemia[2]	717 (8.5)	593 (9.1)
Diverticulosis with bleeding[2]	30 (0.4)	24 (0.4)
Hemorrhage of anus and rectum[2]	282 (3.3)	220 (3.4)
Unspecified gastrointestinal bleedings[2]	606 (7.2)	455 (7.0)
Acute abdominal pain[2]	1,239 (14.7)	1,391 (21.3)
Faecal abnormalities[2]	456 (5.4)	393 (6.0)
Abnormal weight loss[2]	229 (2.7)	189 (2.9)
Irritable bowel syndrome[2]	126 (1.5)	139 (2.1)
Family history of CRC[1]	79 (0.9)	124 (1.9)
Other abnormalities[2,3]	557 (6.6)	549 (8.4)
Any of the above	3,343 (39.6)	3,060 (46.9)

[1] Within the same quarter or ever before diagnosis.
[2] Within the same quarter or the quarter before diagnosis.
[3] Other abnormalities include nausea and vomiting, belching, flatulence and meteorism.

Supplementary Table 2. Proportion of advanced CRCs among all sdCRCs, among sdCRCs with codes for symptoms/ selected diagnoses1, among those without such codes and among non-sdCRCs, stratified by age group.

	Men			Women		
	Mode of detection			Mode of detection		
	sdCRCs		non-sdCRCs	sdCRCs		non-sdCRCs
	Without codes of symptoms/ diagnoses	With codes of symptoms/ diagnoses		Without codes of symptoms/ diagnoses	With codes of symptoms/ diagnoses	
	n (%)	n (%)	n (%)	n (%)	n (%)	n (%)
**Age at diagnosis**						
55-59 years						
All CRCs	635 (100.0)	404 (100.0)	2,994 (100.0)	557 (100.0)	508 (100.0)	2,825 (100.0)
**Stage at diagnosis**						
Advanced	204 (32,1)	170 (42,1)	1,452 (48,5)	167 (30,0)	180 (35,4)	1,207 (42,7)
60-64 years						
All CRCs	808 (100.0)	470 (100.0)	3,856 (100.0)	492 (100.0)	433 (100.0)	3,424 (100.0)
**Stage at diagnosis**						
Advanced	281 (34,8)	197 (41,9)	1,908 (49,5)	163 (33,1)	175 (40,4)	1,526 (44,6)
65-69 years						
All CRCs	971 (100.0)	591 (100.0)	5,010 (100.0)	577 (100.0)	440 (100.0)	4,391 (100.0)
**Stage at diagnosis**						
Advanced	336 (34,6)	252 (42,6)	2,421 (48,3)	167 (28,9)	192 (43,6)	1,977 (45,0)
70-74 years						

All CRCs	1125 (100.0)	730 (100.0)	6,663 (100.0)	731 (100.0)	622 (100.0)	5,932 (100.0)
**Stage at diagnosis**						
Advanced	359 (31,9)	291 (39,9)	3,151 (47,3)	228 (31,2)	231 (37,1)	2,709 (45,7)
75-79 years						
All CRCs	932 (100.0)	648 (100.0)	6,937 (100.0)	639 (100.0)	544 (100.0)	6,501 (100.0)
**Stage at diagnosis**						
Advanced	309 (33,1)	256 (39,5)	3,024 (43,6)	187 (29,3)	178 (32,7)	2,860 (44,0)
80-84 years						
All CRCs	426 (100.0)	342 (100.0)	4,664 (100.0)	336 (100.0)	331 (100.0)	5,288 (100.0)
**Stage at diagnosis**						
Advanced	138 (32,4)	117 (34,2)	1,936 (41,5)	110 (32,7)	110 (33,2)	2,126 (40,2)
≥ 85 years						
All CRCs	192 (100.0)	158 (100.0)	3,367 (100.0)	127 (100.0)	182 (100.0)	5,736 (100.0)
**Stage at diagnosis**						
Advanced	52 (27,1)	55 (34,8)	1,211 (36,0)	38 (29,9)	50 (27,5)	2,032 (35,4)

[1] sCRCs with codes indicating symptoms or relevant diagnoses (e.g. Crohn’s diseases and/or ulcerative colitis, acute abdominal pain, faecal abnormalities) in the same quarter or before screening colonoscopy/FOBT.

Supplementary Table 3. Proportion of sdCRCs stratified by tumor localization.

	Localization		
	Distal	Proximal	Both/Unknown
	n (%)	n (%)	n (%)
**Overall**			
All CRCs	44,485 (100)	28,536 (100)	9,517 (100)
sdCRCs	9,024 (20.3)	4,603 (16.1)	1,324 (13.9)
**Men**			
All CRCs	25,087 (100)	12,774 (100)	4,061 (100)
sdCRCs	5,594 (22.3)	2,211 (17.3)	627 (15.4)
**Women**			
All CRCs	19,398 (100)	15,762 (100)	5,456 (100)
sdCRCs	3,430 (17.7)	2,392 (15.2)	697 (12.8)

Supplementary Table 4. Proportion of patients with at least one comorbidity among men and women with sdCRCs compared to those with non-sdCRCs, stratified by age group (the age distribution within age groups is described in Supplementary Table 5).

	Men		Women	
	Mode of detection		Mode of detection	
	sdCRCs	non-sdCRCs	sdCRCs	non-sdCRCs
	n (%)	n (%)	n (%)	n (%)
**Age at diagnosis**				
55-64 years				
All CRCs	2,317 (100.0)	6,850 (100.0)	1,990 (100.0)	6,249 (100.0)
**Comorbidities**				
1. Coronary heart disease (medication)	165 (7.1)	761 (11.1)	62 (3.1)	231 (3.7)
2. Heart failure	49 (2.1)	213 (3.1)	28 (1.4)	83 (1.3)
3. Acute myocardial infarction	15 (0.6)	59 (0.9)	4 (0.2)	12 (0.2)
4. Acute stroke	16 (0.7)	63 (0.9)	4 (0.2)	20 (0.3)
Any of the above (1-4)	205 (8.8)	911 (13.3)	84 (4.2)	309 (4.9)
5. COPD	106 (4.6)	397 (5.8)	97 (4.9)	312 (5.0)
6. Severe liver disease	6 (0.3)	26 (0.4)	3 (0.2)	32 (0.5)
7. End-stage renal disease	4 (0.2)	48 (0.7)	6 (0.3)	29 (0.5)
8. Diabetes with end-organ damage	4 (0.2)	57 (0.8)	1 (0.1)	18 (0.3)
Any of the above (1-8)	299 (12.9)	1,275 (18.6)	175 (8.8)	631 (10.1)
9. Immunosuppressant therapy	33 (1.4)	224 (3.3)	42 (2.1)	266 (4.3)
10. HIV therapy	3 (0.1)	14 (0.2)	1 (0.1)	2 (0.0)

11. Hemiplegia	26 (1.1)	147 (2.1)	17 (0.9)	101 (1.6)
12. Dementia	2 (0.1)	39 (0.6)	3 (0.2)	22 (0.4)
Any of the above (1-12)	340 (14.7)	1,508 (22.0)	223 (11.2)	898 (14.4)
13. Therapy with antidepressants	67 (2.9)	240 (3.5)	98 (4.9)	419 (6.7)
14. Therapy with antipsychotics	14 (0.6)	79 (1.2)	24 (1.2)	89 (1.4)
Any of the above (13- 14)	76 (3.3)	300 (4.4)	114 (5.7)	482 (7.7)
15. Liver diseases including chronic viral hepatitis	258 (11.1)	830 (12.1)	159 (8.0)	516 (8.3)
16. Treated diabetes	256 (11.1)	1,027 (15.0)	121 (6.1)	446 (7.1)
17. Antihypertensive treatment	1,074 (46.4)	3,158 (46.1)	685 (34.4)	2,205 (35.3)
18. Lipid-modifying agents	296 (12.8)	948 (13.8)	144 (7.2)	460 (7.4)
19. Asthma	129 (5.6)	331 (4.8)	147 (7.4)	433 (6.9)
20. Obesity	440 (19.0)	1,258 (18.4)	336 (16.9)	1,111 (17.8)
65-74 years				
All CRCs	3,417 (100.0)	11,673 (100.0)	2,370 (100.0)	10,323 (100.0)
**Comorbidities**				
1. Coronary heart disease (medication)	580 (17.0)	2,491 (21.3)	175 (7.4)	1,124 (10.9)
2. Heart failure	191 (5.6)	878 (7.5)	72 (3.0)	504 (4.9)
3. Acute myocardial infarction	30 (0.9)	185 (1.6)	6 (0.3)	70 (0.7)
4. Acute stroke	47 (1.4)	207 (1.8)	13 (0.5)	95 (0.9)
Any of the above (1-4)	705 (20.7)	3,004 (25.7)	230 (9.7)	1,479 (14.3)
5. COPD	220 (6.4)	1,011 (8.7)	155 (6.5)	869 (8.4)
6. Severe liver disease	7 (0.2)	37 (0.3)	3 (0.1)	33 (0.3)
7. End-stage renal disease	24 (0.7)	169 (1.4)	9 (0.4)	89 (0.9)
8. Diabetes with end-organ damage	13 (0.4)	116 (1.0)	4 (0.2)	47 (0.5)
Any of the above (1-8)	871 (25.5)	3,703 (31.7)	372 (15.7)	2,200 (21.3)

9.Immunosuppressant therapy	61 (1.8)	411 (3.5)	70 (3.0)	427 (4.1)
10. HIV therapy	0 (0.0)	14 (0.1)	0 (0.0)	1 (0.0)
11. Hemiplegia	51 (1.5)	330 (2.8)	28 (1.2)	199 (1.9)
12. Dementia	38 (1.1)	240 (2.1)	13 (0.5)	196 (1.9)
Any of the above (1-12)	936 (27.4)	4,099 (35.1)	440 (18.6)	2,656 (25.7)
13. Therapy with antidepressants	58 (1.7)	266 (2.3)	89 (3.8)	547 (5.3)
14. Therapy with antipsychotics	18 (0.5)	74 (0.6)	20 (0.8)	118 (1.1)
Any of the above (13-14)	73 (2.1)	323 (2.8)	102 (4.3)	639 (6.2)
15. Liver diseases including chronic viral hepatitis	441 (12.9)	1,517 (13.0)	264 (11.1)	1,124 (10.9)
16. Treated diabetes	688 (20.2)	2,469 (21.1)	238 (10.0)	1,309 (12.7)
17. Antihypertensive treatment	2,111 (61.8)	7,285 (62.4)	1,270 (53.6)	5,628 (54.5)
18. Lipid-modifying agents	751 (22.0)	2,721 (23.3)	315 (13.3)	1,599 (15.5)
19. Asthma	167 (4.9)	569 (4.9)	158 (6.7)	819 (7.9)
20. Obesity	701 (20.5)	2,305 (19.7)	450 (19.0)	1,947 (18.9)
≥75 years				
All CRCs	2,698 (100.0)	14,967 (100.0)	2,159 (100.0)	17,525 (100.0)
**Comorbidities**				
1. Coronary heart disease (medication)	738 (27.4)	4,950 (33.1)	369 (17.1)	3,984 (22.7)
2. Heart failure	299 (11.1)	2,398 (16.0)	224 (10.4)	2,771 (15.8)
3. Acute myocardial infarction	29 (1.1)	303 (2.0)	15 (0.7)	204 (1.2)
4. Acute stroke	37 (1.4)	366 (2.4)	25 (1.2)	382 (2.2)
Any of the above (1-4)	897 (33.3)	6,182 (41.3)	523 (24.2)	5,715 (32.6)
5. COPD	228 (8.4)	1,622 (10.8)	157 (7.3)	1,523 (8.7)
6. Severe liver disease	0 (0.0)	25 (0.2)	2 (0.1)	28 (0.2)
7. End-stage renal disease	41 (1.5)	368 (2.5)	18 (0.8)	246 (1.4)

8. Diabetes with end-organ damage	19 (0.7)	186 (1.2)	5 (0.2)	127 (0.7)
Any of the above (1-8)	1,045 (38.7)	7,057 (47.2)	631 (29.2)	6,697 (38.2)
9.Immunosuppressant therapy	63 (2.3)	588 (3.9)	68 (3.1)	702 (4.0)
10. HIV therapy	1 (0.0)	4 (0.0)	0 (0.0)	0 (0.0)
11. Hemiplegia	71 (2.6)	575 (3.8)	31 (1.4)	507 (2.9)
12. Dementia	118 (4.4)	1,267 (8.5)	76 (3.5)	1,896 (10.8)
Any of the above (1-12)	1,140 (42.3)	7,879 (52.6)	721 (33.4)	8,056 (46.0)
13. Therapy with antidepressants	40 (1.5)	393 (2.6)	86 (4.0)	1,012 (5.8)
14. Therapy with antipsychotics	3 (0.1)	55 (0.4)	8 (0.4)	118 (0.7)
Any of the above (13-14)	43 (1.6)	438 (2.9)	92 (4.3)	1,098 (6.3)
15. Liver diseases including chronic viral hepatitis	331 (12.3)	1,910 (12.8)	236 (10.9)	1,825 (10.4)
16. Treated diabetes	538 (19.9)	3,303 (22.1)	300 (13.9)	2,810 (16.0)
17. Antihypertensive treatment	1,951 (72.3)	11,139 (74.4)	1,502 (69.6)	12,852 (73.3)
18. Lipid-modifying agents	714 (26.5)	4,069 (27.2)	455 (21.1)	3,353 (19.1)
19. Asthma	156 (5.8)	831 (5.6)	147 (6.8)	1,164 (6.6)
20. Obesity	442 (16.4)	2,460 (16.4)	399 (18.5)	2,892 (16.5)

Supplementary Table 5. Age distribution of men and women with sdCRCs compared to non-sdCRCs.

	Men		Women	
	Mode of detection		Mode of detection	
	sdCRCs	non-sdCRCs	sdCRCs	non-sdCRCs
**Age**				
55-64 years				
Mean	59.9	59.9	59.3	60.0
Median	60.0	60.0	59.0	60.0
65-74 years				
Mean	70.0	70.0	69.9	70.0
Median	70.0	70.0	70.0	70.0
≥75 years				
Mean	79.6	81.0	79.9	82.3
Median	79.0	80.0	79.0	81.0
